# Study on the Nonlinear Behavior and Factors Influencing the Axial Compression of High-Durability Fibrous Concrete Wrapped Steel Tube Composite Members

**DOI:** 10.3390/ma15217603

**Published:** 2022-10-29

**Authors:** Jun Wei, Zhenshan Wang, Yanan Su, Jiayi Han

**Affiliations:** 1 School of Civil Engineering, Suzhou University of Science and Technology, Suzhou 215009, China; 2 School of Civil Engineering and Architecture, Xi’an University of Technology, Xi’an 710048, China

**Keywords:** high-corrosion-resistant concrete, thin-walled steel pipe, spiral stiffening ribs, numerical simulation, parametric analysis, ultimate load capacity

## Abstract

Thin-walled steel pipe concrete has better economic performance, but the problem of local buckling is more prominent with a thin-walled steel pipe; meanwhile, thin-walled steel pipe is more sensitive to the environment and the influence of rusting is more prominent. To solve the above problems, this paper proposes new spiral stiffened rib thin-walled steel pipe concrete laminated members to obtain better force and economic performance. Based on axial compression tests on five forms of composite members, this paper studies the nonlinear behavior of the axial compression of this new type of laminated member and the factors influencing it. The following conclusions are obtained. Under the constraint of the spiral ribs, the new composite member has good integrity and each part can ensure cooperative stress; the buckling of the steel pipe is well limited and the mechanical performance is significantly improved. Compared with ordinary thin-walled concrete-filled steel tubular members, the bearing capacity is increased by about 20% and the deformation ability is increased by more than 30%. The nonlinear behavior of the member in compression can be better achieved through finite element analysis. The parametric analysis shows that the pitch and the steel tube width-to-thickness ratio greatly influence the force behavior of the member. In contrast, the spiral rib width-to-thickness ratio and the external reinforcement only need to meet the structural requirements. Finally, based on the superposition theory, the proposed method of calculating the member’s axial compressive load-bearing capacity is given and design suggestions are made. The results of this paper can provide some basis for the engineering application of this new combination member.

## 1. Introduction

Steel pipe concrete is widely used in high-rise, super-high-rise, and large-span structures, due to its high strength and good seismic and fire-resistance properties. The circular section has the best restraint effect on the core concrete and performs significantly better than other section forms in all aspects [1]. However, due to economic issues, the application of this combination member with better performance is basically in a blank state among structures with low vertical loads, such as medium- and low-rise buildings. To solve this problem, scholars have proposed thin-walled concrete-filled steel tubes. The wall thickness of the steel tube is greatly reduced, which can significantly reduce the amount of structural steel and improve the utilization rate of materials to obtain better economic benefits. However, after the steel pipe is thinned, the local stability of the component deteriorates and the steel pipe undergoes buckling deformation in advance so that the core concrete loses the confinement effect of the steel pipe. The ductility of the entire component deteriorates. Even brittle failure occurs [2]. At the same time, when the wall thickness of the steel pipe is reduced, the component is more sensitive and its fire resistance and corrosion resistance will be significantly reduced [3]. Therefore, through technical research and development, it is of high engineering application value to solve the problems of local buckling, poor fire resistance, and corrosion caused by thin-walled steel pipes.

In engineering, plate-stiffening ribs are often used to make thin-walled steel pipe concrete columns less likely to buckle in certain places. Guo [4], Liu [5], Shekastehband [6], and others carried out experimental research on stiffened, thin-walled concrete-filled steel tubular members. The results show that the longitudinal stiffeners are welded on the inner side of the tube wall; they can play a specific supporting role, enhance the friction and embedding effect between concrete and stiffeners, and improve the bending stiffness of the cross-section to delay the local buckling of the steel tube, inhibit the cracking of the concrete, and improve the ductility of components. Zhou [7] and others conducted related research on diagonal stiffeners. The results show that the stiffeners can enhance the steel pipe’s local buckling resistance and improve the bearing and deformation capacity but have little effect on the lateral deformation of the steel pipe. Lu [8] et al. proposed a T-shaped CFST column with oblique honeycomb stiffeners. The study found that setting 45° oblique stiffeners compared with other angle specimens can improve the initial stiffness and slow down the later stiffness degradation. By applying loads in different directions, it is found that the installation of diagonal stiffeners can improve the seismic performance of the specimen on asymmetric sections.

Due to the thin-wall thickness, thin-walled CFST is more sensitive to lateral deformation. In response to this problem, some scholars have proposed CFST composite members with lateral constraints. Ge [9], Ho [10], and others carried out experimental studies on the compressive performance of steel pipe concrete members with restrained ties and found that: the tensile effect of the ties can effectively reduce the radial stress set acting on the steel pipe, increase the average lateral binding force of the steel pipe on the core concrete, and improve the compressive strength of the core concrete, thereby delaying the lateral deformation of the member and improving the ductility and load-bearing capacity of the member. The study also found that the axial spacing of the tie increases the diameter of the tie; adding the number of rows of ties, the more prominent the role of the tie is in restraining the outer drum deformation of the steel pipe [11,12].

In response to the poor deformation capacity of thin-walled steel pipe concrete, some scholars have proposed the setting of spiral hoop reinforcement, circumferential steel plates, and other forms of restraint. The study found that the set of spiral reinforcement can enhance steel pipe concrete’s restraint effect and ultimate bearing capacity. Unlike traditional stiffening ribs, built-in spiral reinforcement does not need to be welded and the construction is more convenient [13,14]. The external steel ring restricts the lateral deformation of the member through the compression effect provided by the steel ring, thus, enhancing the load-bearing capacity and deformation of the member. However, the outer ring plate has a particular impact on the surface flatness of the member and is not as apparent as the vertical stiffening ribs in terms of restraint effect [15,16].

In addition, the integrity of thin-walled steel tube concrete is worse than that of ordinary members. When the steel tube buckles, the debonding between the steel tube and the concrete will significantly weaken the restraint effect of the steel tube [17,18]. To improve the mechanical properties of the interface between steel tube and concrete, scholars have proposed a PBL stiffening-rib structure. Through research, it has been found that PBL stiffening rib, through the embedding effect with concrete, can effectively delay the separation between steel tube wall and concrete and significantly improve the shear capacity and stiffness of the interface [19,20]. On this basis, J.P. Liu [21] and J. Guo [22] et al. studied the compressive behavior of different concrete-filled steel tubular columns. The results show that PBL stiffeners greatly influence the crack distribution of core concrete and can significantly improve the flexural stiffness of members. The flexural bearing capacity is the most significant when the reinforcement hole spacing is two-times the aperture [23]. Scholars [24,25] conducted a numerical simulation study on the static bending performance of beam and plate bridges with connectors based on the first-order shear deformation theory. They revealed the influence law of parameters, such as structural geometry and material properties, on the mechanical response of the structure. Nguyen [26], Tran et al. [27] presented sandwich functionally graded beam high-temperature static bending and thermal buckling based on the third-order shear deformation theory. New results were also found that adding stiffeners to the FGM plate can reduce the weight of the FGM plate, but whether or not stiffeners are provided does not affect the strength of the FGM plate under the same boundary conditions and compressive loads.

This paper proposes a new restraint form of spiral stiffeners based on vertical and spiral stirrups, combining their advantages. The stiffeners are distributed spirally along the outer wall of the steel pipe, which can restrain the thin-walled steel pipe’s local buckling, lateral deformation, and integrity from improving the components’ mechanical performance. At the same time, the exterior is filled with concrete and spiral stiffeners are used to restrain it, which can effectively ensure the cooperative work between the concrete and the core components. When the steel pipe is wrapped with concrete, the internal components’ fire resistance and corrosion resistance can be improved, the later maintenance treatment can be avoided, and the engineering applicability can be improved. As a high-durability material, basalt-fiber-reinforced concrete can enhance the resistance to ion penetration. Using basalt-fiber-reinforced concrete wrapped steel tube can effectively enhance internal components’ fire resistance and corrosion resistance, avoiding post-maintenance treatment and improving engineering applicability. In this paper, based on the axial compression test of the component, the numerical simulation of the nonlinear behavior of compression is carried out and the influence of critical parameters, such as pitch, spiral rib width–thickness ratio, and steel tube width–thickness ratio, is analyzed. Finally, based on the superposition theory, combined with the results of parameter analysis, a new method for calculating the ultimate bearing capacity of composite members is proposed. The research results of this paper can provide a basis for the engineering application of this component.

## 2. Overview of Axial Pressure Test

### 2.1. Test Introduction

**Figure 1:**
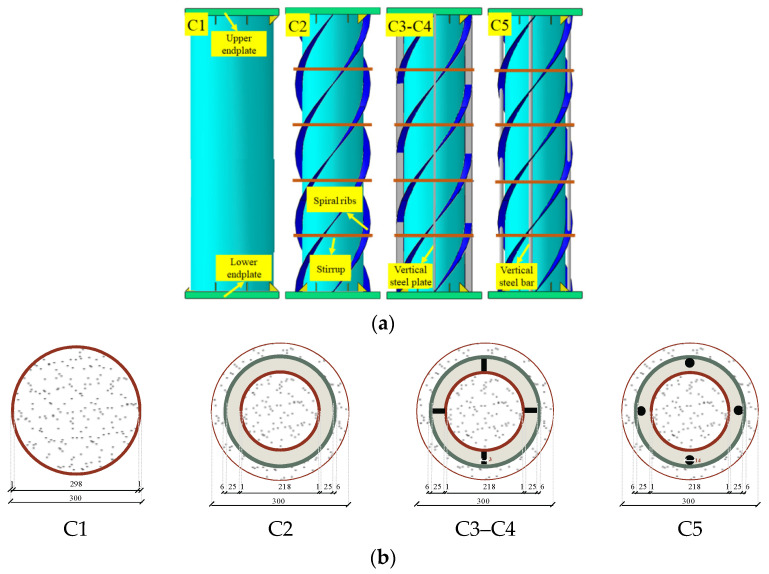
Specimen shape. (**a**) Schematic diagram (**b**) Cross-section form.

This new type of component belongs to the concrete-filled steel tubular composite component from the structural and mechanical point of view, so it is designed according to the “Technical specification for steel tube-reinforced concrete column structure, CECS 188: 2005” [28]. A total of five cross-section forms was designed for the test. The steel pipe diameters were 300 mm and 200 mm, respectively. The specific form is shown in Figure 1 and the specimen parameters are shown in Table 1. The steel of the specimens is Q235-B and the thickness of the steel tube is 1 mm. The stiffeners are welded obliquely in sections to form a spiral distribution with a pitch of 1000 mm, core concrete in C30 self-compacting concrete, external high-durability basalt concrete. The specimen is completed by welding end plates at both ends and leaving a 100 mm hole in the center of the end plate to fill with concrete. The compressive strength of concrete is measured by ‘Standard for test method of mechanical properties on ordinary concrete’ (GB/T 50081-2002) [29] and the steel performance is measured by ‘Metallic materials—Tensile testing—Part 1:Method of the test at room temperature’ (GB/T 228.1-2010) [30]. Specific material properties are shown in Table 2 and Table 3.

**Figure 2:**
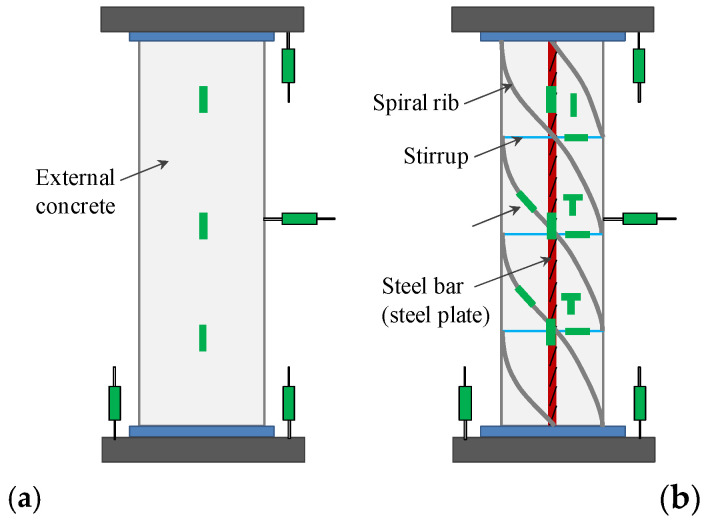
Displacement meter and strain gauge arrangement. (**a**) External concrete (**b**) Steel pipe.

**Figure 3:**
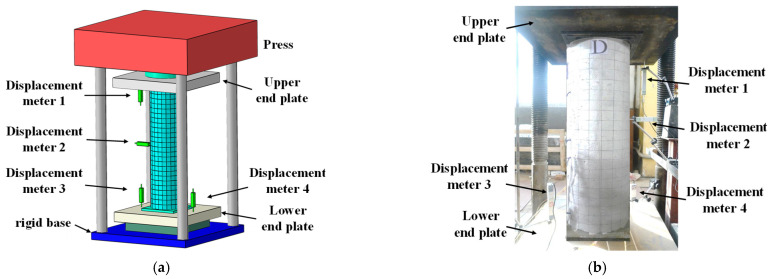
Loading device. (**a**) Simulated loading device (**b**) Test site conditions.

The specimen displacement meter and strain gauge arrangement are shown in Figure 2 and the loading device is shown in Figure 3. Graded loading was used. The initial stage was load controlled (50 kN/min) and 75% of the ultimate load was reached to shift to displacement control, with a loading rate of 0.1 mm/min, and the test ended when the load was reduced to 85% of the peak load.

### 2.2. Experimental Process

**Figure 4:**
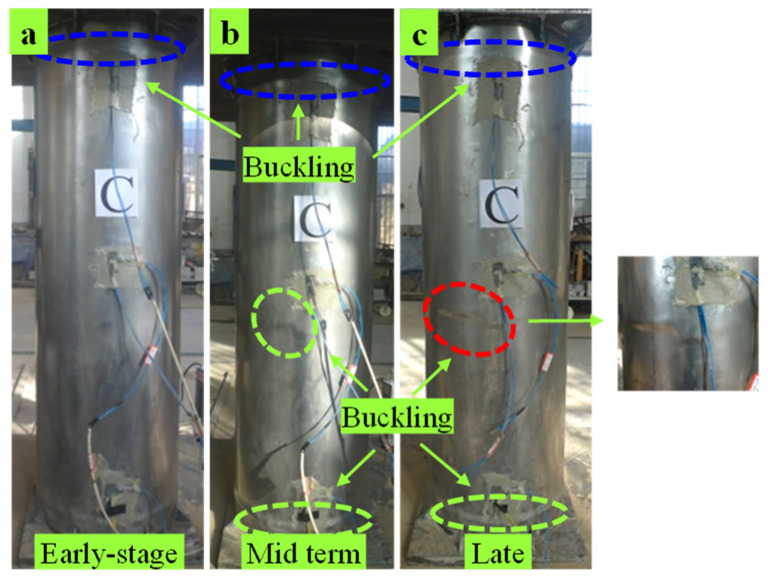
Damage phenomenon of C1. (**a**) Early-stage (**b**) Mid term (**c**) Late.

C1 specimen: For the initial loading, when loaded to 1350 kN, the specimen from the upper-end plate had 80 mm slight buckling, as shown in Figure 4a, and for sustained loading, buckling extended to form corrugated ring buckling; when loaded to an ultimate load of around 1925.8 kN, the upper and lower ends of the column are connected and developed and the buckling is about 8–10 mm, as shown in Figure 4b; as the load continues to decline, the buckling gradually extends to the lower part of the column, accompanied by the sound of the collision of the inner concrete with the pipe wall. When the load is reduced to 1420 kN, the buckling of each part of the steel tube is distributed in a large area and the buckling of the upper, lower, and middle parts of the column is the most serious. The final failure mode of the specimen is shown in Figure 4c.

**Figure 5:**
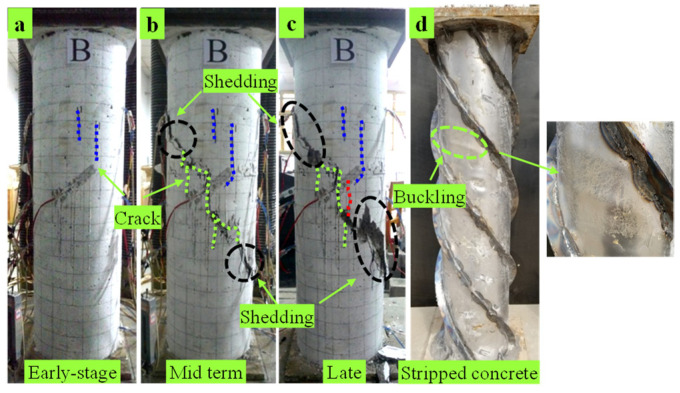
Damage phenomenon of C2. (**a**) Early-stage (**b**) Mid term (**c**) Late. (**d**) Stripped concrete.

C2 specimen: At the initial stage of loading, several vertical cracks at 200 mm from the upper-end plate are shown in Figure 5a; for continuous loading, the upper cracks extend obliquely to the adjacent surface of the specimen; in the middle stage of loading, the cracks on each surface of the specimen continue to expand along the direction of spiral ribs. When the load reaches 1832.5 kN, the cracks extend to the middle and lower parts of the specimen and the width extends to 5 mm with concrete peeling, as shown in Figure 5b. In the later loading stage, the crack width of each surface of the specimen is up to 20 mm and the concrete falls off along the inclined crack position in the direction of the spiral rib and the failure mode is shown in Figure 5c. As shown in Figure 5d, there is buckling of about 5 mm in the upper part of the steel tube and there is no evident buckling in the remaining parts.

**Figure 6:**
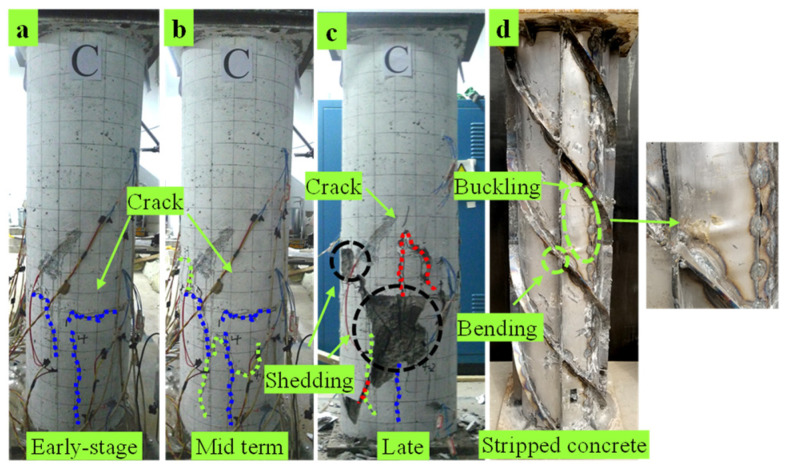
Damage phenomenon of C3. (**a**) Early-stage (**b**) Mid term (**c**) Late. (**d**) Stripped concrete.

C3 specimen: At the initial loading stage, when the load increases to 971.9 kN, the vertical micro-cracks extending downward appear in the middle of the specimen. With continuous loading, vertical cracks appeared in the middle and lower parts of each specimen surface, as shown in Figure 6a. When the load reached 1925.2 kN, transverse and longitudinal intersecting cracks appeared at 200 mm from the bottom of the specimen, accompanied by the sound of concrete crushing, as shown in Figure 6b. In the later stage of loading, a large area of concrete fell off along the direction of spiral ribs and the oblique cracks of adjacent surfaces and the failure mode is shown in Figure 6c. The concrete outside the specimen is peeled off, the middle of the steel tube bulges, and the vertical plate has severe bending, as shown in Figure 6d.

**Figure 7:**
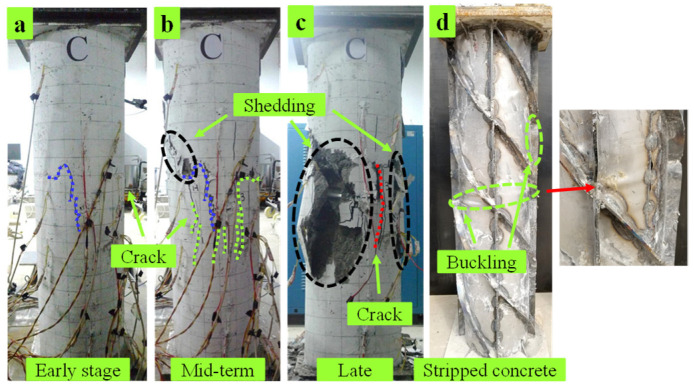
Damage phenomenon of C4. (**a**) Early-stage (**b**) Mid term (**c**) Late. (**d**) Stripped concrete.

C4 specimen: In the early stage of loading, oblique downward cracks appeared in the middle of the specimen along the direction of spiral ribs, as shown in Figure 7a; when the load continued to increase to 1774.2 kN, multiple vertical cracks began to appear in the middle. In the middle stage of loading, each vertical crack extends downward to 8 mm in width, accompanied by concrete peeling and small pieces falling off, as shown in Figure 7b. In the later stage of loading, all the peeled concrete around the cracks fell off, the middle part of the cracks along the spiral ribs bulged seriously, and the specimen was destroyed, as shown in Figure 7c. Stripping the outer-cladding concrete revealed that a bulge appeared in the middle of the steel pipe, the vertical plate was bent and deformed, and the change in the spiral rib was not noticeable, as in Figure 7d.

**Figure 8:**
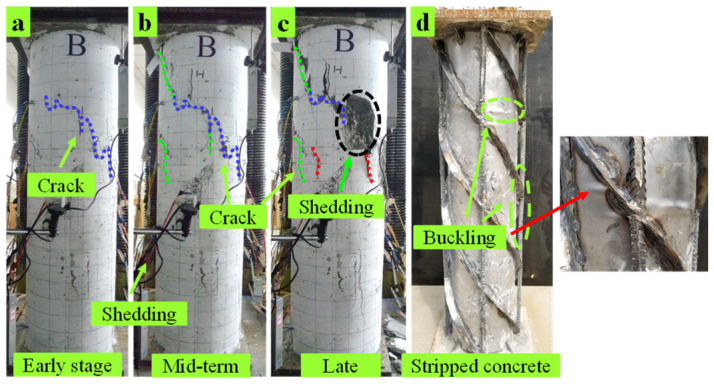
Damage phenomenon of C5. (**a**) Early-stage (**b**) Mid term (**c**) Late. (**d**) Stripped concrete.

C5 specimen: In the early loading stage, oblique cracks appeared in the upper part of the compression zone (C surface), as shown in Figure 8a. When the load increased to 1720 kN, fine cracks appeared on all surfaces. When loading to the middle stage, the original crack extended upward and downward along the direction of the spiral ribs. Diagonal cracks were generated in the middle, as shown in Figure 8b. In the later stage of loading, the concrete in the middle and upper parts of the compression zone fell off in large pieces along the direction of the spiral rib and the vertical cracks intersected with the oblique cracks, with a comprehensive development depth and the specimen, finally failed, as shown in Figure 8c. When the concrete was stripped, we found that the upper part of the steel tube appears to have a small range of buckling, steel, and spiral rib bending deformation, as shown in Figure 8d.

### 2.3. Load-Displacement Curve

**Figure 9:**
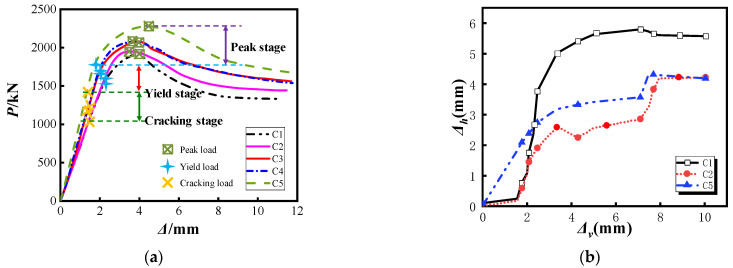
Specimen load–displacement curve and transverse deformation. (**a**) Load–displacement curve (**b**) Steel tube deformation comparison diagram.

The comparison of load–displacement curves of each specimen is shown in Figure 9a and the comparison of load–displacement eigenvalues is shown in Table 4. It can be seen from the figure that, compared with ordinary components, the stiffness of the specimen with helical rib restraint is generally more significant at the initial stage of loading. With the load increase, the specimen enters the elastic–plastic stage, at which time the specimen is transformed from the individual stress of each part to the cooperative stress working state. Among them, the C5 specimen has the best mechanical performance under the double constraints of spiral ribs and vertical steel bars. Compared with the single spiral rib constraint specimen, the bearing capacity of the reinforced column is increased by 17.4% and the deformation capacity is increased by 18.6%. The reason is that after welding vertical reinforcement, the broad steel bearing area of the C5 column is 2.5-times larger than that of the C2 column, significantly improving the ultimate bearing capacity and ductility. In addition, the descending section of specimens C3 and C4 is the most gentle and the deformation capacity is better, which is 20.7% higher than that of the C1 column, indicating that the vertical plate has a good effect on resisting deformation.

Figure 9b shows the buckling displacement measured by displacement meter 2 in the middle of the steel tube. The figure shows that the middle of the steel tube of specimen C1 has the most significant degree of bulging, with bulging displacement close to 6 mm. After the single spiral rib (C2) is set, the lateral displacement of the steel tube is reduced and the maximum is about 4 mm, indicating that the spiral rib has a particular effect on suppressing the circumferential deformation of the steel tube. The maximum displacement of the C5 specimen (spiral rib-steel bar) is the same as that of the C2 specimen, which shows that the addition of a steel bar does not affect the effect of transverse restraining deformation and the combination of the two mainly plays the role of improving ductility.

## 3. Finite Element Simulation

### 3.1. Modelling

Based on the spiral rib vertical-reinforcement-restrained steel tube concrete stacked column with the most superior force performance in the axial compression test, a numerical model was established using ABAQUS to perform finite element analysis on the stacked column [31].

#### 3.1.1. Steel Constitutive Relation

In this test, steel pipes, spiral ribs, vertical steel plates, and stirrups are all made of Q235 grade steel and HRB400 steel bars are used for vertical steel bars. In the finite element simulation process, steel, as elastic–plastic material, obeys the Von Mises yield criterion. Ref. [32] selected the constitutive relation of steel. The stress–strain relationship is divided into five stages: elastic stage (OA), elastic–plastic stage (AB), plastic stage (BC), strengthening stage (CD), and secondary plastic flow (DE), with the following expressions (the relevant parameters are taken by the results of the material properties tests):

(1) $$\sigma_{s}=\left\{\begin{array}{l} E_{s}\varepsilon_{e}\\ -A\varepsilon_{e}^{2}+B\varepsilon_{e}+C\\ f_{y}\\ f_{y}\left[1+0.6\frac{\varepsilon_{e}-\varepsilon_{e2}}{\varepsilon_{e3}-\varepsilon_{e2}}\right]\\ 1.6f_{y} \end{array}\right.\;\;\;\begin{array}{c} \varepsilon \leq \varepsilon_{e}\\ \varepsilon_{e}<\varepsilon \leq \varepsilon_{e1}\\ \varepsilon_{e1}<\varepsilon \leq \varepsilon_{e2}\\ \varepsilon_{e2}<\varepsilon \leq \varepsilon_{e3}\\ \varepsilon \geq \varepsilon_{e3} \end{array}$$

where: $\varepsilon_{e}=0.8f_{y}/E_{s}$; $\varepsilon_{e1}=1.5\varepsilon_{e}$; $\varepsilon_{e2}=10\varepsilon_{e1}$; $\varepsilon_{e3}=100\varepsilon_{e1}$; $A=0.2f_{y}/{\left(\varepsilon_{e1}-\varepsilon_{e}\right)}^{2}$, $B=2A\varepsilon_{e1}$; $C=0.8f_{y}+A{\left(\varepsilon_{e}\right)}^{2}-B\varepsilon_{e}$.

#### 3.1.2. Concrete Principal Structure Relationships

The concrete of concrete-filled steel tubular columns includes core concrete and outer concrete, both of which adopt the plastic damage model of concrete.

1. Stress–strain relationships for core concrete

The core concrete is subjected to three-dimensional compression due to the confinement of the steel tube and its strength and deformation capacity are significantly improved. When selecting the constitutive relationship, the confinement effect of the steel tube should be considered. This paper uses the constitutive model proposed by Tao Zhong et al. [33]. The calculation formula of the stress–strain relationship of concrete under compression is as follows:

(2) $$\sigma =\left\{\begin{array}{l} f_{c}^{\prime }\left(\frac{F\cdot X+G\cdot X^{2}}{1+\left(F-2\right)X+\left(G+1\right)X^{2}}\right)\;\;\;\;\;\;\;\;\;\;\;\;\;\;\;\;\;\left(0<\varepsilon \leq \varepsilon_{c0}\right)\\ f_{c}^{\prime }\;\;\;\;\;\;\;\;\;\;\;\;\;\;\;\;\;\;\;\;\;\;\;\;\;\;\;\;\;\;\;\;\;\;\;\;\;\;\;\;\;\;\;\;\;\;\;\;\;\;\;\left(\varepsilon_{c0}\leq \varepsilon <\varepsilon_{\mathrm{cc}}\right)\\ f_{r}+\left(f_{c}^{\prime }-f_{r}\right)\exp\left[-{\left(\frac{\varepsilon -\varepsilon_{\mathrm{cc}}}{\alpha }\right)}^{\beta }\right]\;\;\left(\varepsilon \geq \varepsilon_{\mathrm{cc}}\right) \end{array}\right.$$

Among them: $X=\frac{\varepsilon }{\varepsilon_{c0}}$, $F=\frac{E_{c}\varepsilon_{c0}}{f_{c}^{\prime }}$, $G=\frac{{\left(F-1\right)}^{2}}{0.55}-1$, $\varepsilon_{c0}=0.00076+\sqrt{\left(0.626f_{c}^{\prime }-4.33\right)\times 10^{-7}}$, $\frac{\varepsilon_{\mathrm{cc}}}{\varepsilon_{c0}}=e^{k}$, $k=\left(2.9224-0.00367f_{c}^{\prime }\right){\left(\frac{f_{B}}{f_{c}^{\prime }}\right)}^{0.3124+0.002f_{c}^{\prime }}$, $f_{B}=\frac{\left(1+0.027f_{y}\right)\cdot e^{-0.02\frac{D}{t_{s}}}}{1+1.16e^{-10}\cdot {\left(f_{c}^{\prime }\right)}^{4.8}}$, $f_{r}=0.7\left(1-e^{-1.38\xi }\right)f_{c}^{\prime }\leq 0.25f_{c}^{'}$, $\alpha =0.04-\frac{0.036}{1+e^{6.08\xi -3.49}}$. where ξ is a dimensionless value to measure the restraining effect of the steel pipe on the core concrete, which is calculated by the following equation considering the restraining situation of the laminated members in this paper.

(3) $$\xi =A_{s1}f_{y1}+A_{s2}f_{y2}/A_{c}f_{c}^{'}$$

2. Stress–strain relationships for encased basalt fibrous concrete

According to ‘Code for design of concrete structures’ [34], select:

When $\varepsilon_{c}\leq \varepsilon_{0}$:

(4) $$\sigma_{c}=f_{c}\left[1-{\left(1-\frac{\varepsilon_{c}}{\varepsilon_{0}}\right)}^{n}\right]$$

When $\varepsilon_{0}\leq \varepsilon_{c}\leq \varepsilon_{cu}$:

(5) $$\sigma_{c}=f_{c}$$

Among them, $\varepsilon_{0}=0.002$, $\varepsilon_{cu}=0.0033$, *n* is the correlation coefficient (taken as 2.0 when greater than 2.0).

### 3.2. Grid Division and Interactions

About the finite element simulation of spiral rib-vertical steel bar confined circular concrete filled steel tubular column, the hexahedral mesh model is obtained by controlling the mesh size and using the structural optimization technique to mesh. Reducing mesh transition and improving mesh quality by neutral axis algorithm, the operation convergence is achieved by adjusting the mesh density and load steps.

For the spiral rib vertical-reinforcement-restrained circular steel pipe concrete column, its core concrete, outer-cladding concrete, and upper- and lower-end slabs are simulated by C3D8R solid unit, and thin-walled steel pipe and spiral rib are simulated by S4R shell unit. The T3D2 truss unit simulates vertical reinforcement and hoop for finite element simulation.

Define the interaction between the components: steel pipe and concrete for the surface contact, the average direction using “hard contact”; that is, do not consider the concrete and steel pipe bonding slip effect, tangential contact surface friction coefficient is taken as 0.3, the outer surface of the concrete is the main surface, the outer surface of the steel pipe is from the surface; the upper- and lower-end plates and the inner and exterior concrete, the spiral rib, and the steel pipe, the spiral rib and the vertical steel bar are all bound and restrained; the outer concrete and the spiral rib and the vertical steel bar are all embedded restraint; the upper- and lower-end plates are in contact with the helical rib and steel pipe by shell-solid coupling.

**Figure 10:**
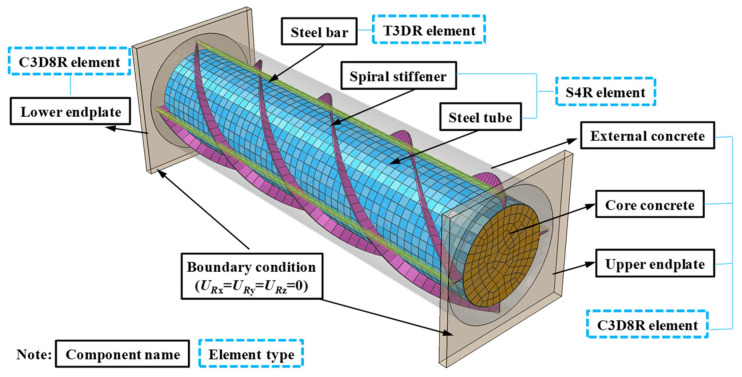
Finite element model diagram.

According to the specific conditions of the axial compression test, the load and boundary conditions are set: the upper- and lower-end plates are regarded as rigid end plates, two reference points are placed at the end plates, and coupling constraints are established with the top surface of the specimen; the displacement load is applied through the coupling points, see Figure 10 for the specific model.

### 3.3. Finite Element Model Validation Analysis

**Figure 11:**
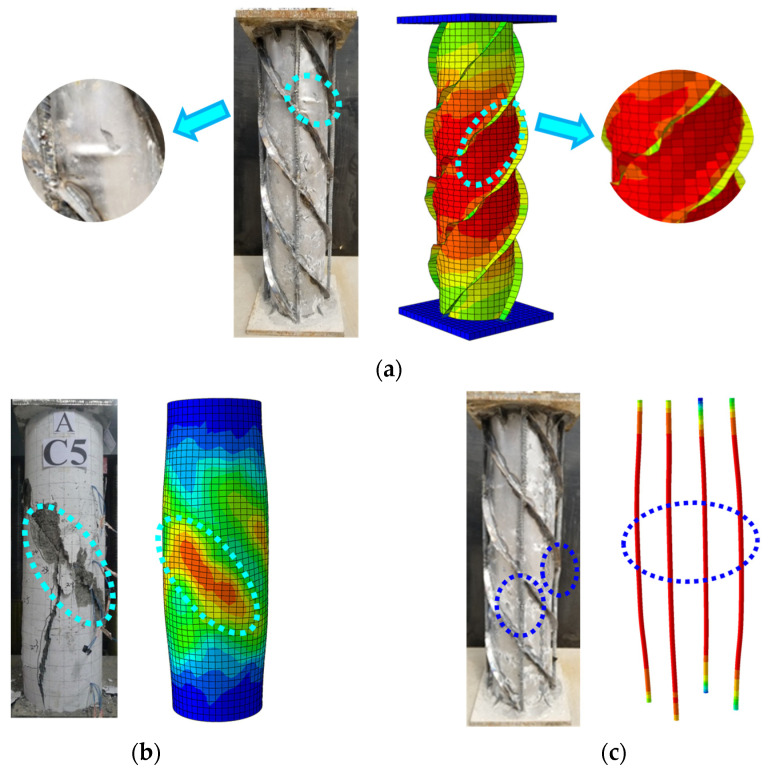
Comparison of test phenomena and simulations. (**a**) Spiral ribbed steel pipes (**b**) External concrete (**c**) Steel reinforcement.

The finite element analysis found that the local buckling of the steel pipe occurred between the spiral ribs, which is consistent with the test situation, as shown in Figure 11a. In the finite element model, cracking occurred in the middle of the exterior concrete along the diagonal 45°, forming a plastic damage zone and, in the test, the external concrete occurred along the spiral rib direction of diagonal cracking, the damage pattern of the two matches, see Figure 11.

**Figure 12:**
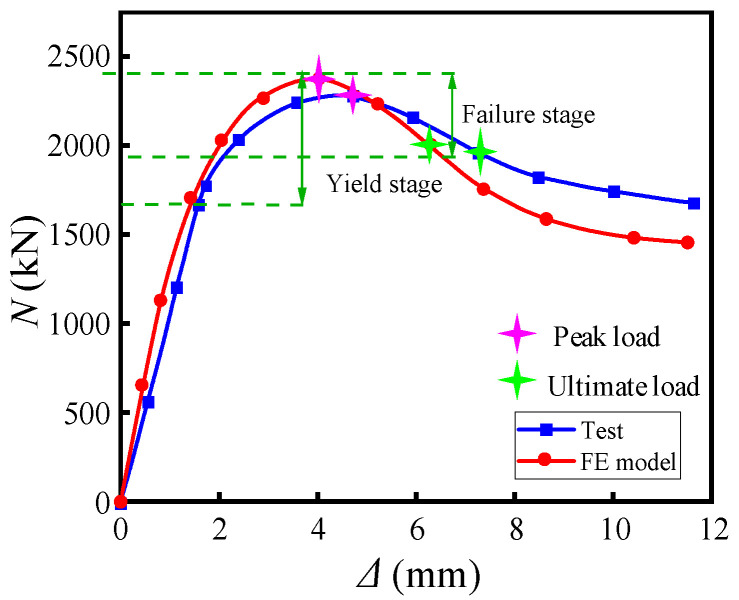
Comparison of load–displacement curves.

Figure 12 compares the load–displacement curves of the test and the finite element simulation. The analysis shows that the finite element model’s stiffness and initial load capacity are slightly more significant than the test’s. The error is around 3%, which is a good match; the model curve decreases faster in the later stage. In summary, the model can better achieve the axial compression force performance of reinforced-spiral rib thin-walled steel pipe concrete columns.

## 4. Parametric Analysis

Based on the finite element verification model, considering the influence of the width–thickness ratio of the spiral rib, the width–thickness ratio of the steel pipe, and the pitch and the diameter of the vertical steel bar, the parameter analysis of the model is carried out. The specific parameters are shown in Table 5.

### 4.1. Width–Thickness Ratio of Spiral Rib

**Figure 13:**
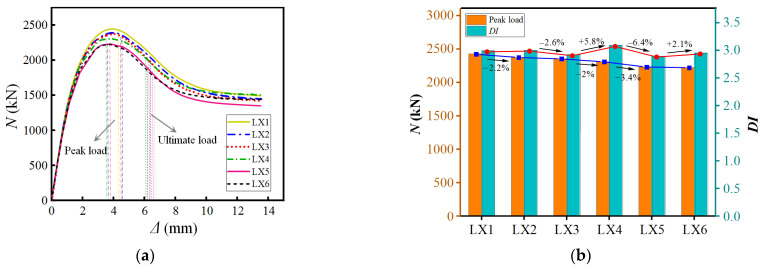
Different spiral rib width–thickness ratio. (**a**) Load–displacement curve (**b**) Performance Changes Note: *DI*: Ductility factor.

Parameter analysis of different spiral rib width–thickness ratios and load–displacement comparison is shown in Figure 13. It is observed that the change in the width–thickness ratio has no noticeable effect on the improvement in bearing capacity. As the width–thickness ratio increases, the bearing capacity of the component increases by about 4%. When the width of the spiral rib is constant, the bearing capacity decreases slightly with the increase in thickness. The reason is that the width of the spiral rib is constant and the increase in the thickness has little effect on the buckling of the steel tube. However, it is not conducive to the integrity of the external concrete and the core member. The concrete is more likely to crack, resulting in a decrease in bearing capacity. Overall, the spiral rib has little contribution to the vertical bearing capacity of the component, mainly to restrain the steel tube’s local buckling and lateral deformation, to meet the bending stiffness required to limit buckling. It is suggested that the width-to-thickness ratio of spiral ribs is 6.5~10.

### 4.2. Width–Thickness Ratio of Steel Pipe

**Figure 14:**
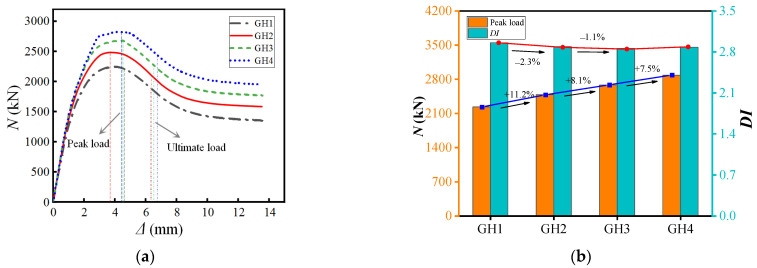
Different width–thickness ratio of steel pipe. (**a**) Load–displacement curve (**b**) Performance Changes.

Figure 14 compares load–displacement curves under different steel tube width–thickness ratios. The analysis found that the greater the wall thickness of the steel tube (i.e., the smaller the width-to-thickness ratio), the more pronounced the load-bearing capacity enhancement effect of the member. The analysis is due to the thickening of the tube wall to enhance the confinement effect of the steel tube on the core concrete so that the specimen has better deformation capacity. Compared with the specimen with a thickness of 1 mm, the bearing capacity of the steel tube with a thickness of 2.5 mm increased by 29.2%, and the ductility decreased by 2.6%. At the same time, it is observed that when the wall thickness of the steel tube increases to 2 mm, the thickness of the steel tube continues to increase and the bearing capacity and ductility of the component are not significantly improved. Based on the comprehensive analysis, the recommended width–thickness ratio of the steel pipe is 100~150.

### 4.3. Pitch

**Figure 15:**
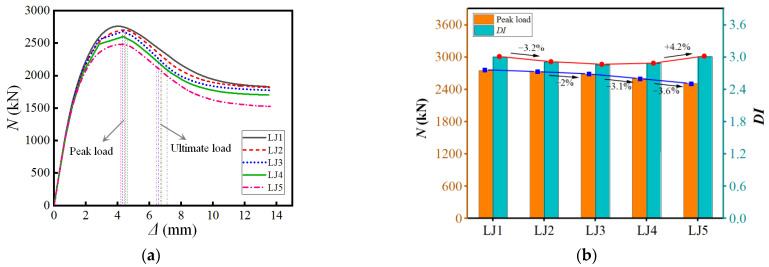
Different pitch. (**a**) Load–displacement curve (**b**) Performance Changes.

The load–displacement curves under five pitches are compared, as shown in Figure 15. The analysis found that the bearing capacity and deformation capacity of the component with a pitch of 500 are relatively weak. As the pitch increases, the bearing capacity of the specimen increases. Compared with the pitch size of 500, the bearing capacity of the specimen with a pitch of 1500 is enhanced by 9.6%. In summary, the larger the pitch, the more pronounced the improvement in the vertical stiffness and ductility of the component; on the contrary, the smaller the pitch, the more pronounced the lateral restraint effect on the steel tube, thereby limiting the buckling of the steel tube and slowing down the failure rate of the specimen. It is suggested that the pitch of the spiral rib should be between 1*H* and 1.4*H*.

### 4.4. Bar Diameter

**Figure 16:**
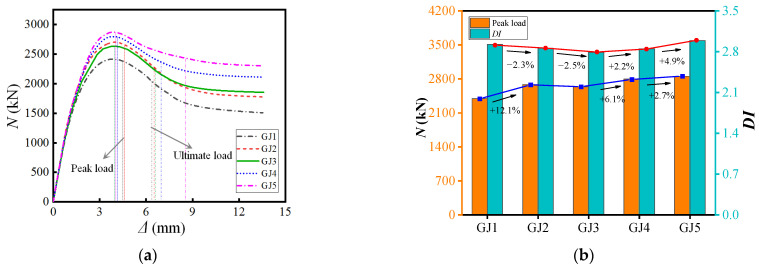
Different bar diameters. (**a**) Load–displacement curve (**b**) Performance Changes.

The parameter analysis of the diameter of the vertical steel bar is carried out and the load–displacement comparison is shown in Figure 16. The results show that the diameter of the steel bar dramatically influences the overall bearing capacity and ductility of the component. Compared with the rebar diameter of 10 mm, when the diameter increased to 14 mm, the bearing capacity of the component increased by 12.1%; when it increased to 25 mm, the bearing capacity increased by 19% and the ductility increased by 2.2%. However, a steel bar diameter that is too large or too small will impact concrete cracking; therefore, as an external concrete construction measure, it is recommended that the spacing is not greater than 200 mm. The diameter of reinforcement $d_{i}$ was taken from 9/100–3/25 $B_{s}$.

## 5. Calculation of Load-Carrying Capacity

**Figure 17:**
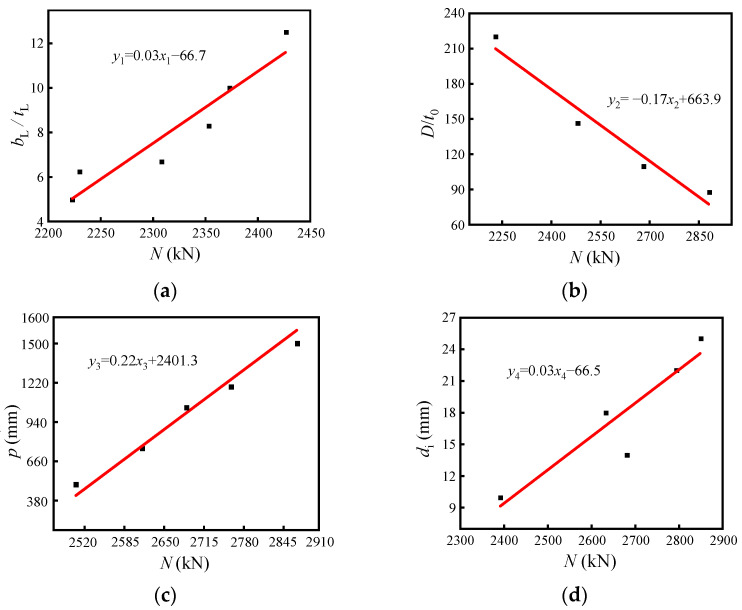
Fitting pattern of each parameter. (**a**) Width–thickness ratio of Spiral Rib (**b**) Width–thickness ratio of Steel Pipe (**c**) Pitch (**d**) Bar Diameter.

The ultimate load-carrying capacity of this combined member is analyzed using superposition theory [35]. It consists of four main components: core concrete load-carrying capacity (*N*_1_), reinforcement load-carrying capacity (*N*_2_), spiral rib steel pipe load-carrying capacity (*N*_3_), and external concrete (*N*_4_). Among them, the core concrete is restrained by the spiral rib-steel pipe and its bearing capacity is improved. The core concrete enhancement factor under the sleeve restraint φ is introduced to measure its reinforcement effect, φ-value calculation method: substitute the load capacity from the finite element simulation into Equation (14) to obtain the φ-value. The restraint effect of steel on core concrete for steel pipe concrete sections can be assessed by the constraint factor of the spiral rib steel tube γ, where η is the comprehensive influence factor of steel tube strength. By fitting the law in Figure 17, η is calculated according to Equation (7).

(6) $$\gamma =\frac{\left(A_{s1}\times f_{y1}+A_{s2}\times f_{y2}\right)\times \left(1+\eta \right)}{A_{c1}f_{\mathrm{ck}1}}$$

(7) $$\eta =0.03b_{L}/t_{L}-0.17D/t_{0}+0.22p+0.03d_{i}$$

**Figure 18:**
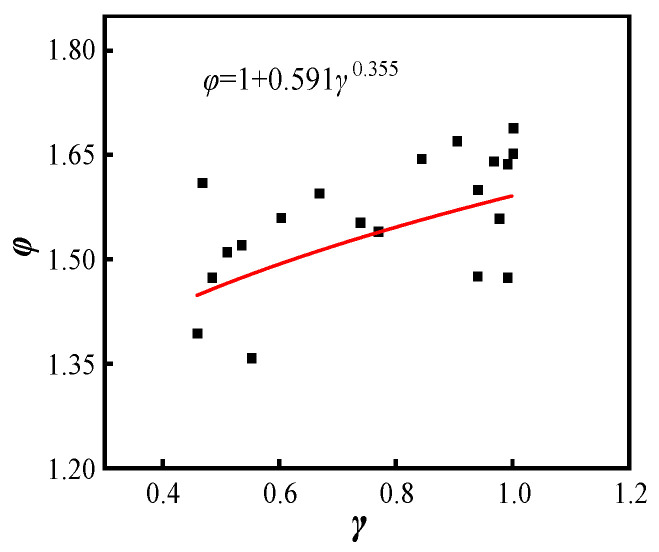
γ fitting pattern.

The values of φ and γ for the different parameter components are obtained and the two satisfy the following law by fitting the curve (Figure 18).

(8) $$\varphi =1+0.591\gamma^{0.355}$$

Therefore, the core concrete bearing capacity *N*_1_ is calculated as follows.

(9) $$N_{1}=\varphi \times A_{c1}\times f_{\mathrm{ck}1}$$

The reinforcement bearing capacity *N*_2_ is calculated according to the following formula.

(10) $$N_{2}=A_{s3}\times f_{y3}$$

For spiral rib steel pipe bearing capacity, consider the new spiral rib form of restraint when the impact of thin-walled steel pipe stabilizes, for the introduction of spirally ribbed steel pipe bearing capacity impact coefficient ω. The coefficient ω is related to the steel pipe width–thickness ratio $D/t_{0}$, spiral rib width–thickness ratio $b_{L}/t_{L}$ and pitch p, where ω with p, $b_{L}/t_{L}$ increases and increases with $D/t_{0}$, which increases and decreases, according to the finite element simulation results, fitting to get its impact law curve; specifically, see Figure 17 and take its slope as a spiral rib steel pipe impact factor, to obtain ω calculation formula.

(11) $$\omega =0.03b_{L}/t_{L}-0.17D/t_{0}+0.22p$$

Spiral ribbed steel pipe bearing capacity *N*_3_ contains two parts, spiral rib and thin wall steel pipe, which are calculated as follows.

(12) $$N_{3}=\omega \times (f_{y1}\times A_{s1}+f_{y2}\times A_{s2})$$

For external concrete in the loading by the lateral deformation of the steel pipe and spiral rib extrusion and other factors, the strength will occur a certain degree of reduction due to this part of the bearing capacity and its compressive strength and cross-sectional area are related. Therefore, for the introduction of the external concrete strength reduction factor λ, according to the test value of 0.8, the formula is as follows.

(13) $$N_{4}=\lambda \times A_{c2}\times f_{ck2}$$

According to the superposition theory, the *N*_u_ bearing capacity of this superimposed column is calculated as follows.

(14) $$\begin{array}{l} N_{u}=N_{1}+N_{2}+N_{3}+N_{4}\\ =\varphi \times A_{c1}\times f_{\mathrm{ck}1}+A_{s3}\times f_{y3}+\omega \times (f_{y1}\times A_{s1}+f_{y2}\times A_{s2})+\lambda \times A_{c2}\times f_{ck2} \end{array}$$

Comparing the above equations with the finite element results shows that the error is within 10%, a high degree of accuracy, as shown in Table 6, which provides a basis for applying this new laminated component.

## 6. Conclusions

In this paper, based on the axial compression test of this new composite member, the nonlinear analysis of the compressive performance and the study of the influence law were carried out and the following conclusions were obtained.

(1) The buckling of the steel tube in the new composite member occurs between the spiral ribs and the external concrete cracks obliquely along the spiral ribs. Compared with ordinary thin-walled steel tube concrete, the bearing capacity is increased by 20% and the deformation capacity is increased by more than 30%.

(2) Spiral rib, as a new form of restraint, can better limit the local buckling of the steel pipe, reduce the lateral deformation, and limit the external concrete cracking to ensure all parts of the force synergy.

(3) The finite element model of this laminated column can better realize its compressive nonlinear behavior and the parameter analysis shows that: the pitch and steel tube width–thickness ratio has the most significant influence on the bearing capacity of the specimen. Design suggestions are given: spiral rib width–thickness ratio ranges from 6.5 to 10; steel pipe width-thickness 100 to 150; pitch 1.0*H* to 1.4*H*; Diameter of reinforcement $d_{i}$ taken from 9/100–3/25 $B_{s}$.

(4) Based on the results of the parametric analysis, the influence law of four parameters, such as the width–thickness ratio of the spiral rib, is given by fitting and, based on the superposition theory, the calculation formula for the axial compressive load capacity of this new laminated member is proposed.

## Figures and Tables

**Table 1:** Parameters of the specimens.

Specimen	Constraint Form	*D* × *t*_0_ (mm)	*H* (mm)	*b*_L_ × *t*_L_ (mm)	*d*_i_ (mm)
C1	-	300 × 1	1000	-	-
C2	Spiral ribs	220 × 1	1000	25 × 3	14
C3	Spiral ribs + Steel plate	220 × 1	1000	25 × 3	14
C4	Spiral ribs + Steel plate (welded)	220 × 1	1000	25 × 3	14
C5	Spiral ribs + Steel bar	220 × 1	1000	25 × 3	14

Note: *D* × *t*_0_: Steel tube diameter and thickness; *H*: Specimen heigh; *b*_L_ × *t*_L_: Spiral rib width and thickness; *d*_i_: Diameter of vertical steel bar.

**Table 2:** Material properties of concrete.

Concrete Grades	*f_cu,k_* (MPa)	*f_ck_* (MPa)	*f_c_* (MPa)	*E_c_* (GPa)
Core plain concrete	37.10	24.81	17.72	25.4
Exterior clad basalt fibrous concrete	34.42	23.02	16.44	24.5

**Table 3:** Steel material properties.

Type	*f_y_* (MPa)	*f_u_* (MPa)	*f_y_*/*f_u_*	*E* (10^5^ MPa)	*δ* %
steel tube (1 mm)	278.70	388.93	0.72	2.06	27.3
stiffening rib (3 mm)	256.94	363.79	0.71	2.03	25.7
Steel plate (5 mm)	252.65	359.26	0.71	2.02	22.7
Steel bar (Φ14)	462.95	625.30	0.73	1.96	24.6

**Table 4:** Load–displacement eigenvalues.

Specimen	*N_cr_* (kN)	*N_y_* (kN)	*N_max_* (kN)	*N_u_* (kN)	Δ*_y_*(mm)	Δ*_max_*(mm)	Δ*_u_*(mm)	Δ*_u_*/Δ*_y_*	Δ*_u_*/Δ*_max_*
C1	-	1540.6	1925.8	1636.9	2.16	4.03	5.22	2.42	1.30
C2	1184	1638.2	1945.6	1653.8	2.03	3.88	6.79	3.34	1.75
C3	971.9	1672.0	2090.0	1776.5	1.90	3.91	7.61	4.01	1.95
C4	1239.8	1669.8	2087.3	1774.2	1.90	3.82	7.66	4.03	2.01
C5	1532	1827.8	2284.7	1942.0	1.82	4.23	7.21	3.96	1.70

**Table 5:** Model parameters.

Specimen	*b*_L_ (mm)	*t*_L_ (mm)	*b*_L_/*t*_L_	*D* (mm)	*t*_0_ (mm)	*D*/*t*_0_	*P* (mm)	*d*_i_ (mm)
LX1	25	2	12.5	220	1	220	1000	14
LX2	30	3	10.0	220	1	220	1000	14
LX3	25	3	8.3	220	1	220	1000	14
LX4	20	3	6.7	220	1	220	1000	14
LX5	25	4	6.25	220	1	220	1000	14
LX6	15	3	5.0	220	1	220	1000	14
GH1	25	4	6.25	220	1	220	1000	14
GH2	25	4	6.25	220	1.5	147	1000	14
GH3	25	4	6.25	220	2	110	1000	14
GH4	25	4	6.25	220	2.5	88	1000	14
LJ1	25	4	6.25	220	2	110	1500	14
LJ2	25	4	6.25	220	2	110	1200	14
LJ3	25	4	6.25	220	2	110	1000	14
LJ4	25	4	6.25	220	2	110	800	14
LJ5	25	4	6.25	220	2	110	500	14
GJ1	25	4	6.25	220	2	110	1000	10
GJ2	25	4	6.25	220	2	110	1000	14
GJ3	25	4	6.25	220	2	110	1000	18
GJ4	25	4	6.25	220	2	110	1000	22
GJ5	25	4	6.25	220	2	110	1000	25

Note: $b_{L}$: Spiral rib width; $t_{L}$: Thickness of spiral rib; $b_{L}/t_{L}$: Spiral rib width-to-thickness ratio; D: Diameter of steel tube; $t_{0}$: Thickness of steel tube Steel tube diameter and thickness; $D/t_{0}$: Steel tube diameter to thickness ratio; p: Pitch; $d_{i}$: Diameter of vertical steel bar. Specimen name definition: LX1–LX6: Variation in spiral rib width-to-thickness parameter; GH1–GH4: Variation in steel pipe width-to-thickness parameters; LJ1–LJ5: Variation in pitch parameters; GJ1–GJ5: Variation in rebar diameter parameters.

**Table 6:** Comparison of calculation results.

Specimen Number	γ	ϕ	Simulated Values *N* (kN)	Calculated Values *N*_u_ (kN)	Error %
LX1	0.47	1.45	2426.4	2278.6	6.49
LX2	0.53	1.47	2372.6	2327.8	1.93
LX3	0.51	1.47	2352.8	2309.5	1.87
LX4	0.48	1.46	2307.9	2291.1	0.74
LX5	0.55	1.48	2230.3	2339.8	4.68
LX6	0.46	1.45	2223.4	2272.3	2.15
GH1	0.55	1.48	2230.2	2339.8	4.68
GH2	0.77	1.54	2483.5	2481.1	0.10
GH3	0.93	1.59	2680.0	2623.8	2.14
GH4	0.99	1.64	2881.4	2770.2	4.01
LJ1	0.99	1.59	2750.9	2647.4	3.91
LJ2	0.99	1.59	2731.7	2641.6	3.41
LJ3	0.98	1.59	2680.4	2635.5	1.70
LJ4	0.97	1.59	2602.2	2626.3	0.92
LJ5	0.94	1.58	2509.8	2602.3	3.55

## Data Availability

Data are contained within the article.

## Nomenclature

H	Specimen heigh	F,G	Dimensioning factor
$b_{L}$	Spiral rib width	$E_{c}$	Modulus of elasticity for concrete
$b_{B}$	Vertical steel plate width	$E_{s}$	Steel elastic modulus
$t_{L}$	Thickness of spiral rib	$f_{y}$	Steel yield strength
$t_{B}$	Vertical steel plate thickness	$f_{u}$	Ultimate strength of steel
$d_{i}$	Diameter of vertical steel bar	$f_{y}/f_{u}$	Steel yield ratio
$B_{s}$	Spacing of bars	$f_{y1}$	Steel tube yield strength
p	Pitch	$f_{y2}$	Yield strength of spiral stiffener
$f_{y3}$	Reinforcement yield strength	$\varepsilon_{c0}$	Peak strain of unconstrained core concrete under compression
δ	Elongation	$\varepsilon_{\mathrm{cc}}$	Peak strain of confined core concrete under compression
$f_{\mathrm{cu},k}$	Standard value of compressive strength of concrete cubes	ξ	Hoop coefficient of concrete filled steel tube
$f_{c,k}$	Standard value of axial compressive strength of concrete	$A_{s1}$	Cross-sectional area of steel tube
		$A_{s2}$	Cross-section area of spiral rib
		$A_{s3}$	The cross-section area of vertical steel bar
$f_{c}$	Design value of compressive strength of outsourced concrete	$A_{c1}$	Cross-sectional area of core concrete
		$A_{c2}$	The cross-sectional area of outsourcing concrete
$f_{\mathrm{ck}1}$	Standard value of compressive strength of core concrete	$N_{\mathrm{cr}}$	Cracking load
		$N_{y}$	Yield load
$f_{\mathrm{ck}2}$	Standard value of compressive strength of outsourced concrete	$N_{\max}$	Peak axial load
$f_{B}$	Residual stress	$N_{u}$	Ultimate load
$f_{r}$	Side limiting stress	$N_{1}$	Core concrete bearing capacity
		$N_{2}$	Steel bar bearing capacity
		$N_{3}$	Bearing capacity of spiral rib steel tube
		$N_{4}$	Outsourcing concrete bearing capacity
$D/t_{s}$	Steel pipe diameter to thickness ratio	$\Delta_{y}$	Cracking displacement
$\sigma_{s}$	Steel stress	$\Delta_{\max}$	Displacement corresponding to peak load
$\sigma_{c}$	Exterior concrete compressive stress	$\Delta_{u}$	Displacement corresponding to ultimate load
$\varepsilon_{0}$	Strain of Encased Concrete When Compressive Stress Reached Design Value	$\Delta_{u}/\Delta_{y}$	Deformability
$\varepsilon_{c}$	Compressive strain of outsourcing concrete	$\Delta_{u}/\Delta_{\max}$	Emergency capacity
$\varepsilon_{\mathrm{cu}}$	The ultimate compressive strain of normal section concrete	γ	Constraint factor of spiral rib steel tube
φ	Core concrete enhancement factor under sleeve restraint	ω	Spirally ribbed steel pipe bearing capacity impact coefficient
η	Comprehensive influence factor of steel tube strength	λ	External concrete strength reduction factor
